# Strategies for mapping synaptic inputs on dendrites *in vivo* by combining two-photon microscopy, sharp intracellular recording, and pharmacology

**DOI:** 10.3389/fncir.2012.00101

**Published:** 2012-12-13

**Authors:** Manuel Levy, Adrien E. Schramm, Prakash Kara

**Affiliations:** Department of Neurosciences, Medical University of South CarolinaCharleston, SC, USA

**Keywords:** intracellular recording, cat visual cortex, dendrite, calcium imaging, two-photon imaging, spike suppression, GABA iontophoresis, QX-314

## Abstract

Uncovering the functional properties of individual synaptic inputs on single neurons is critical for understanding the computational role of synapses and dendrites. Previous studies combined whole-cell patch recording to load neurons with a fluorescent calcium indicator and two-photon imaging to map subcellular changes in fluorescence upon sensory stimulation. By hyperpolarizing the neuron below spike threshold, the patch electrode ensured that changes in fluorescence associated with synaptic events were isolated from those caused by back-propagating action potentials. This technique holds promise for determining whether the existence of unique cortical feature maps across different species may be associated with distinct wiring diagrams. However, the use of whole-cell patch for mapping inputs on dendrites is challenging in large mammals, due to brain pulsations and the accumulation of fluorescent dye in the extracellular milieu. Alternatively, sharp intracellular electrodes have been used to label neurons with fluorescent dyes, but the current passing capabilities of these high impedance electrodes may be insufficient to prevent spiking. In this study, we tested whether sharp electrode recording is suitable for mapping functional inputs on dendrites in the cat visual cortex. We compared three different strategies for suppressing visually evoked spikes: (1) hyperpolarization by intracellular current injection, (2) pharmacological blockade of voltage-gated sodium channels by intracellular QX-314, and (3) GABA iontophoresis from a perisomatic electrode glued to the intracellular electrode. We found that functional inputs on dendrites could be successfully imaged using all three strategies. However, the best method for preventing spikes was GABA iontophoresis with low currents (5–10 nA), which minimally affected the local circuit. Our methods advance the possibility of determining functional connectivity in preparations where whole-cell patch may be impractical.

## Introduction

**Figure 1:**
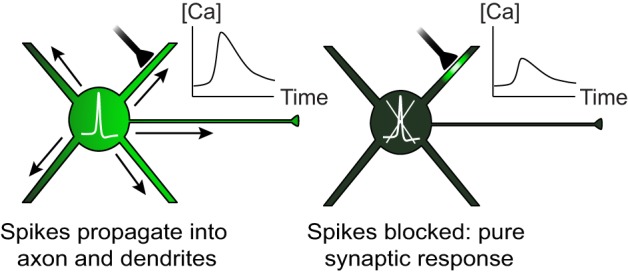
**Both local synaptic inputs and spikes initiated at the soma can cause dendritic calcium transients.** When spikes are blocked, dendritic calcium can be used as a reporter of local synaptic activity.

To understand how the neocortex processes sensory information, we need to establish the relationship between the functional properties of individual neurons and the wiring diagram that connects them together. One of the main obstacles to this goal is that sensory responses can only be measured *in vivo* whereas neuronal connectivity is typically mapped *in vitro*. By gaining access to the subthreshold membrane potential, *in vivo* intracellular recordings partially bridged this gap and greatly improved our understanding of the synaptic underpinnings of sensory receptive fields (Borg-Graham et al., 1998; Bringuier et al., 1999; Hirsch et al., 2003; Priebe and Ferster, 2005; Wehr and Zador, 2005; Higley and Contreras, 2006; Tan et al., 2011). However, intracellular recordings alone cannot disentangle the functional properties of individual synapses distributed across the dendritic tree. Recent progress in optical imaging techniques has created new opportunities to examine the functional connectivity of cortical neurons (Jia et al., 2010; Marshel et al., 2010; Bock et al., 2011; Chen et al., 2011; Ko et al., 2011; Rancz et al., 2011; Varga et al., 2011). In particular, the Konnerth group has been able to successfully image dendritic sensory responses of putative synaptic origin in layer 2/3 pyramidal neurons *in vivo*, across several areas of the mouse neocortex (Jia et al., 2010; Chen et al., 2011; Varga et al., 2011, also see Svoboda et al., 1997, 1999; Helmchen et al., 1999; Takahashi et al., 2012). Individual neurons were labeled with a fluorescent calcium-sensitive dye through a patch pipette before imaging evoked responses in dendrites with two-photon microscopy. These sensory-evoked calcium signals were then used to infer the functional properties of presynaptic inputs and analyze their organization on the dendritic tree. Importantly, to ensure that the dendritic calcium signals are of synaptic origin, spikes must be prevented from back-propagating into the dendrites (Figure 1). This was achieved by injecting current through the patch electrode to hyperpolarize the neuron below spike threshold.

To date, the mouse is the only mammalian species where dendritic synaptic inputs have been imaged *in vivo*. However in primates, cats and ferrets, the primary visual cortex (V1) is organized into precise maps for a variety of stimulus attributes, e.g., orientation, direction, ocular dominance, and binocular disparity (Grinvald et al., 1986; Hubener et al., 1997; Ohki et al., 2005; Kara and Boyd, 2009). These feature maps may be associated with wiring strategies that are different from those used in rodent V1.

Unfortunately, stable long-lasting patch recordings with simultaneous sub-cellular imaging *in vivo* are more challenging in large mammals. Significant brain pulsations are associated with respiration and heartbeat. The cortical tissue may also be more opaque under two-photon microscopy in non-rodent mammals like cats. Moreover the feline arachnoid and pia are relatively thick and tough, so a large positive pressure is needed to keep the tip of the patch electrode clear of cellular debris. Large positive pressure is impractical, because it may lead to an accelerated accumulation of fluorescent dye in the extracellular space, which will reduce the calcium indicator signal-to-noise ratio in dendrites (Jia et al., 2011). Thus not as many whole-cell recordings may be attempted at a given cortical site in cats compared to rodents. Together, these limitations make imaging the dendrites of whole-cell patched neurons very difficult in the non-rodent mammalian brain.

As an alternative to whole-cell patch, a sharp intracellular microelectrode can be used to load the calcium indicator into the neuron and record the membrane potential (Jaffe et al., 1992; Svoboda et al., 1997, 1999; Helmchen et al., 1999). Intracellular recordings *in vivo* can be obtained in cat V1 using both techniques, but typically last longer with sharp than with patch electrodes (Borg-Graham et al., 1998; Bringuier et al., 1999). Moreover, due to the small tip diameter of the sharp pipette, dye leakage in the extracellular milieu is negligible. Thus, several attempts at intracellular recording are possible in the same cortical region of interest without changing the electrode. Once intracellular access has been gained, the neuron can be loaded quickly by applying current of the appropriate sign (Svoboda et al., 1997, 1999; Helmchen et al., 1999). However, due to the large impedance of sharp electrodes, even with good quality recordings (high input resistance) it may be difficult to inject sufficient current to hyperpolarize the membrane potential below spike threshold during a barrage of sensory-evoked EPSPs.

In the present study, we evaluated three different strategies to prevent spiking in neurons loaded with a calcium indicator via a sharp electrode: (1) hyperpolarization by injection of negative current, (2) pharmacological intracellular blockade of voltage-gated sodium channels with QX-314, and (3) near-somatic ejection of GABA from an iontophoresis electrode glued to the sharp intracellular electrode. The last strategy currently appears to be the most promising tool for mapping the functional connectivity in the neocortex of non-rodent mammals.

## Materials and methods

### Animal preparation

**Figure 2:**
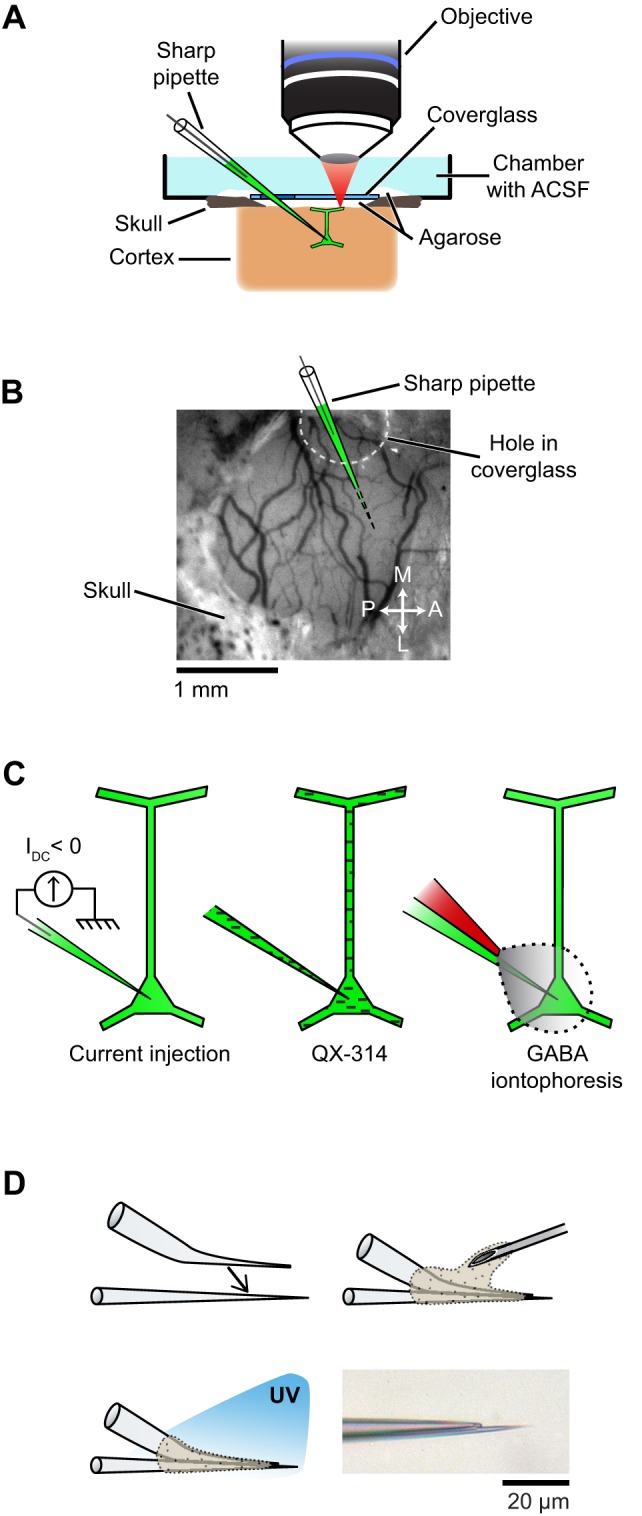
**Setup and experimental strategies used to prevent spikes during functional mapping of synaptic inputs *in vivo*. (A)** Schematic of the preparation showing simultaneous intracellular recording and dendritic calcium imaging in a layer 2/3 pyramidal neuron *in vivo*. **(B)** The electrode enters the cortex through a hole in the coverglass. **(C)** Spikes are suppressed either by injection of a constant negative current to hyperpolarize the neuron below spike threshold (left), or by including QX-314 in the pipette solution (middle), or by iontophoresis of GABA from an adjacent patch pipette glued to the sharp microelectrode (right). **(D)** Fabrication of the double barrel electrode used for simultaneous intracellular recording and extracellular iontophoresis of GABA: a bent patch pipette and a straight sharp microelectrode are bonded together by UV-sensitive glue. Bottom right, micrograph of the two tips of a completed double barrel electrode (inter-tip distance: 15 μm). Also see Figure A4 in Appendix for detailed steps on the assembly of double barrel electrodes.

**Figure A1:**
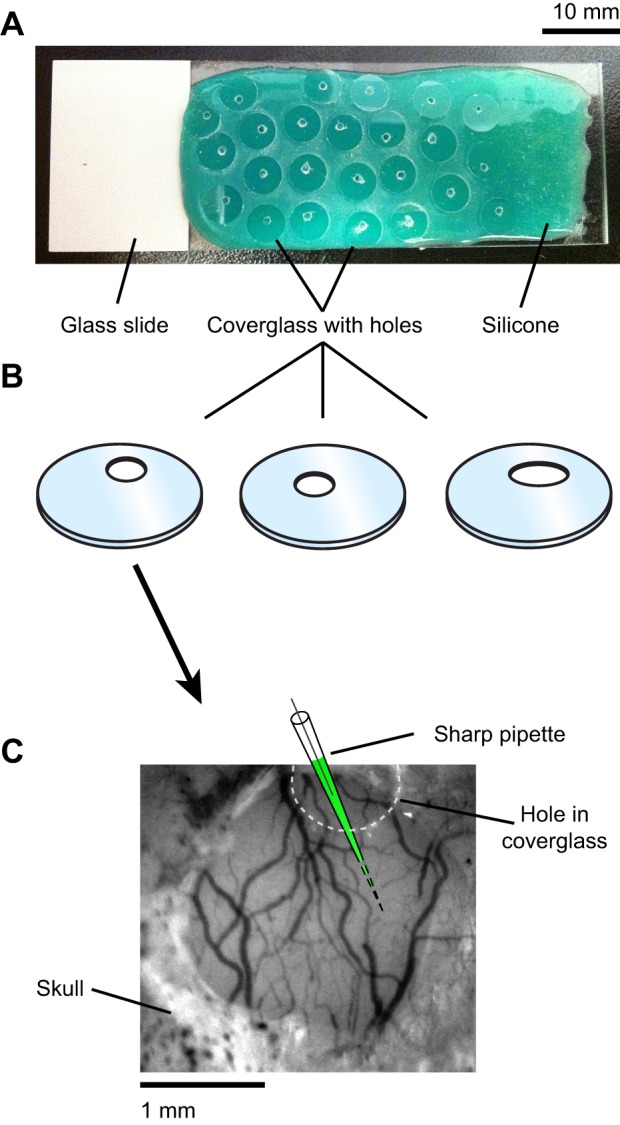
**Drilling holes in the coverglass for electrode access to the craniotomy. (A)** Standard rectangular glass slides were first coated with a layer of fast-curing silicone elastomer (Kwik–Cast, World Precision Instruments). Individual circular coverglasses were then placed on the silicone surface of the slide. Holes varying in shape and location were drilled into the coverglasses using a dental bur (see “Materials and Methods”). Only one hole per coverglass was made. The silicone coating provided mechanical stability and scratch proofing of the coverglass during drilling. **(B)** The coverglasses were separated from the silicone and cleaned with ethanol. **(C)** One coverglass was selected such that the position and shape of the hole allowed electrode access to an imaging site free of large blood vessels on the cortical surface.

Physiological experiments were performed in cats of either sex (postnatal days 28–42). All procedures were approved by the Institutional Animal Care and Use Committee of the Medical University of South Carolina and were based on those previously published (Kara and Boyd, 2009; O'Herron et al., 2012; Shen et al., 2012). Anesthesia was induced by IM injection of ketamine (20 mg/kg) and acepromazine (0.2 mg/kg) and maintained by isofluorane (1–2% during surgery, 0.5–1% during imaging). The depth of anesthesia was assessed throughout the experiment by monitoring the electrocardiogram and the electroencephalogram. The animal was paralyzed with vecuronium bromide (0.2 mg/kg/h, IV) and artificially ventilated through a tracheal cannula. The end tidal CO_2_ was maintained between 3.5% and 4.5%. A heating pad maintained the rectal temperature between 37.0 and 38.0°C. Dexamethasone (1 mg/kg, IM) and atropine (0.1 mg/kg, subcutaneous) were injected to prevent inflammatory reactions and to reduce secretions, respectively. A headplate was cemented to the skull over the primary visual cortex, a 1.5 mm × 3 mm craniotomy was performed and the dura was resected. To reduce heart and respiratory induced pulsations, the underlying cortex was covered with warm agarose (2.5% in ACSF) and the craniotomy was sealed by a coverglass (5 mm diameter, 0.17 mm thick, World Precision Instruments) (see Figure 2A). Additional warm agarose was applied to the edge of the coverglass and adjacent bone for increased stability (Figure 2A). The coverglass had a ~1 mm diameter hole to allow access for the microelectrodes (Figures 2A,B). The hole was pre-drilled in the coverglass before the experiment started by placing the coverglass on a silicone-coated slide and using a carbide #1 (Miltex) dental bur (Figure A1 in Appendix).

### Visual stimulation

Drifting (2 Hz, 25–100% contrast) square- or sine-wave gratings were presented at a variety of orientations, directions of motion, spatial frequencies, spatial positions, binocular phase disparities, and monocularly through the left or right eye. For ocular dominance or binocular disparity assays, each animal viewed monoptic and dichoptic stimuli displayed on a CRT monitor (160 Hz) through custom ultra-fast ferroelectric liquid crystal shutters. All other tests were conducted monocularly through the dominant eye. Visual stimuli were presented for 4–8 s interspersed with equal duration blank periods and the stimulus sequence was repeated 2–10 times.

### Intracellular electrophysiological recording and dye loading

Sharp microelectrodes were pulled from filamented borosilicate glass capillaries (1.5 mm/0.84 mm, outer/inner diameter) using a horizontal micropipette puller (P-97, Sutter Instrument Company). The resistance of the microelectrodes was ~90 MΩ (range 70–110 MΩ) when filled with 2 M potassium methyl sulfate (Acros Organics) and 2–4 mM Oregon Green BAPTA-1 (OGB-1) hexapotassium salt (Life Technologies) at pH = 7.3–7.5. In some cases the pipette solution also contained 20–100 mM QX-314 (Alomone Labs) to block voltage gated sodium channels (Figure 2C) and 200 μM Alexa Fluor 633 hydrazide (Alexa 633) (Life Technologies) to label neurons with a red, calcium-insensitive dye. Intracellular membrane potentials were recorded using an Axoclamp 2B amplifier (Molecular Devices) in bridge mode. Signals were low-pass filtered at 3 kHz and digitized at 10 kHz using Micro1401 hardware and Spike2 software (Cambridge Electronic Design). Electrodes were inserted at a 30° angle and advanced to 200–400 μm below the pia, until a neuron was impaled. Immediately after impalement, negative current was delivered to stabilize the recording and to help the diffusion of the negatively charged OGB-1 into the neuron. The neurons included in our data set had resting membrane potentials of −65.5 ± 10.4 mV, input resistances of 37.7 ± 10.7 MΩ and spike heights of 44.6 ± 8.0 mV (all values mean ± SD). Dendrites were sufficiently labeled for calcium imaging 20–30 min after impalement. We used this loading time to map the preferred orientation, the preferred spatial frequency, the ocular dominance and the receptive field location of the spiking responses.

### Double barrel electrodes and GABA iontophoresis

**Figure A2:**
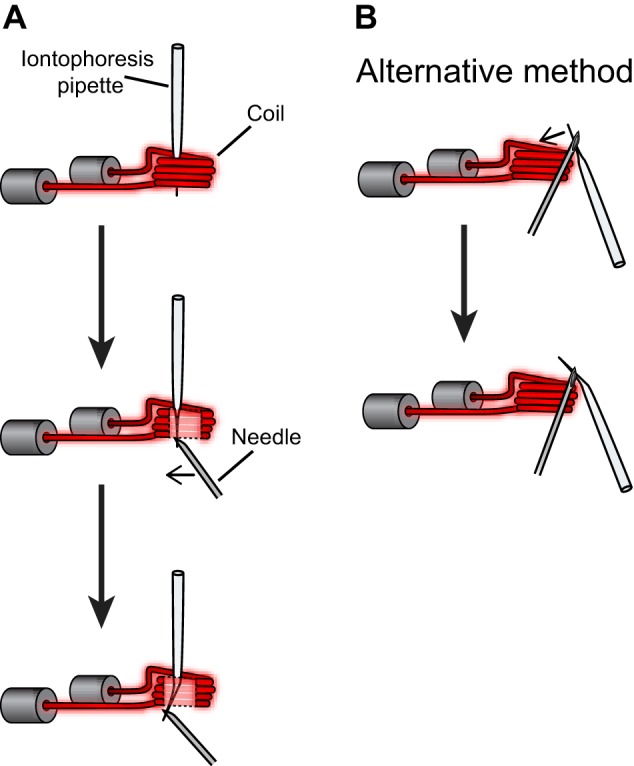
**Procedures for bending the iontophoresis electrode. (A)** The iontophoresis electrode was positioned in the coil of a vertical electrode puller and heated (current intensity ~17 A, solenoid turned off). Then the tip of the electrode was bent by applying pressure with a needle. **(B)** Alternatively the iontophoresis electrode can be held outside the coil and bent by pushing toward the heat source.

**Figure A3:**
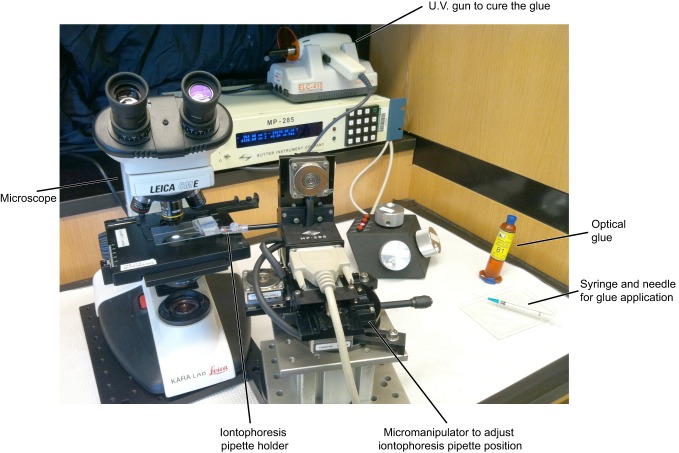
**Overall setup used for the assembly of double barrel electrodes.** The various elements necessary for joining the intracellular sharp and extracellular iontophoresis electrodes are shown (also see Figure A4).

**Figure A4:**
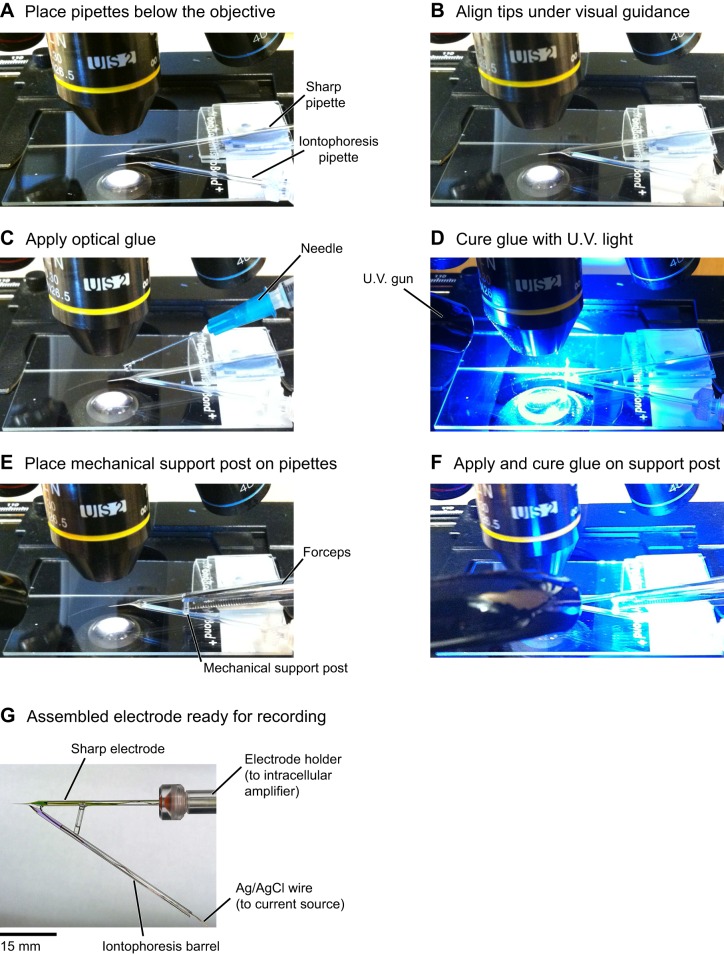
**Detailed steps for the assembly of double barrel electrodes. (A)** The sharp pipette is attached to a glass slide using modeling clay and the iontophoresis pipette is held by the micromanipulator. **(B)** Under visual guidance the pipette shanks are aligned side-by-side, primarily by using the manipulator to move the bent iontophoresis electrode into position. **(C)** A drop of optical glue is carefully applied on the shanks with a needle. Avoiding touching the tips with glue is essential for clog-free operation of the electrodes. **(D)** The glue is cured by shining U.V. light for a couple of minutes. **(E,F)** Gluing a glass capillary transversely between the two electrodes provides mechanical support for the double barrel assembly. **(G)** Completed double barrel electrode. The sharp electrode is loaded with KMS, OGB-1 salt and Alexa 633; the iontophoresis electrode is loaded with GABA and Alexa 594 (see “Materials and Methods”).

Iontophoresis electrodes were pulled using the same type of glass capillaries as for sharp electrodes. The puller program was modified so that the shank length of the iontophoretic electrode was comparable to the sharp electrode (~8–9 mm) and the tip diameter was larger for the iontophoresis electrode (~1 μm) than for the sharp electrode (<0.2 μm). The iontophoresis electrode was bent to an angle of ~30° by pressing on the shank (~5 mm above the tip) with a 16G needle during exposure to the heating filament of a vertical puller (PE-2, Narishige, see Figure A2 in Appendix). The iontophoresis electrode was mounted on an electrode holder connected to a micromanipulator, while the sharp electrode was mounted on a glass slide using molding clay (Figure A3 in Appendix). The tip of the sharp electrode was brought into focus under the 10× objective (UPlanFl N 0.3 NA, 10 mm WD) of a brightfield microscope. Then, the shank of the iontophoresis electrode was brought in close apposition to the shank of the sharp electrode by using the micromanipulator (Figure 2D and Figure A4 in Appendix). The tip of the sharp electrode protruded in front of the tip of the iontophoretic electrode by <20 μm. A thin layer of optical glue (NOA 81, Norland Products) was applied on the shanks with a 23G needle, as close as possible to the tips without clogging them, and cured by exposure to UV light (ELC 410, Electro-Lite Corporation) (Figure A4 in Appendix). The iontophoresis electrode was then filled with 0.5 M GABA (pH ~5.5) and 50 μM Alexa Fluor 594 hydrazide (Alexa 594) (Life Technologies) (Figure A4 in Appendix). Iontophoretic currents were generated by a current source (Microiontophoresis Current Programmer 260, World Precision Instruments). Before placing the double barrel electrode in the cortex, we checked that the two electrodes were not clogged by passing current through each of them. Leakage of GABA during electrode advancement in the cortex was prevented by using retention currents of −8 to −10 nA. Each time we obtained a stable intracellular recording, we determined the minimal ejection current necessary for suppressing the spike responses to optimal visual stimuli. This current, typically +5 to +10 nA, was then applied during functional dendritic imaging.

### Two-photon imaging

#### Hardware

OGB-1 fluorescence was monitored using a custom-built microscope (Prairie Technologies) coupled with a Mai Tai Deep See (Newport Spectra-Physics) mode-locked Ti:sapphire laser (810 nm or 920 nm). Excitation light was focused by a water-immersion objective (Olympus LUMPlan Fl/IR 40×, 0.8 NA, 3.3 mm WD). The average power delivered to the brain was kept below 40 mW. Two non-descanned photomultiplier tube (PMT) detector channels (Hamamatsu R3896) were used with a primary dichroic (700dcxr, Chroma) and IR blocking filter (ET 700 sp-2p8, Chroma), and barrier filters (Chroma) customized for the fluorophores of interest: ET 525/50m-2p for OGB-1, ET 665/40m-2p for Alexa 633, and HQ 645/70m-2p for Alexa 594 (see Shen et al., 2012). Scanning was performed using two 6 mm galvanometer mirrors. After dendritic functional imaging, z-stacks were acquired for *post-hoc* reconstruction of the dendritic tree in Neurolucida software (MBF Bioscience).

#### Intracellular loading of OGB-1 salt for dendritic imaging

Sharp microelectrodes included 2–4 mM Oregon Green BAPTA-1 (OGB-1) hexapotassium salt, as described above (see “Intracellular Electrophysiological Recording and Dye Loading”).

#### Bulk loading of OGB-1 AM for population cell body imaging

Our procedure was adapted from Stosiek et al. (2003) and described in detail elsewhere (Kara and Boyd, 2009; O'Herron et al., 2012; Shen et al., 2012). Briefly, 50 μg of OGB-1 AM (Life Technologies) were dissolved in 4 μl DMSO with 20% pluronic acid F-127 (Sigma). This solution was further diluted to 1:10 with pipette solution (150 mM NaCl, 2.5 mM KCl, 10 mM HEPES, pH 7.4), for a final concentration of 1 mM OGB-1 AM. The solution also included Alexa 594 (50 μM) for pipette visualization. This dye solution was loaded in a patch pipette (tip diameter ~1–2 μm) and pressure ejected (50–90 pulses, 1.0 s pulse duration, 2–10 psi) into the cortex ~200 μm below the pia. After an hour the dye was fully taken up by neurons.

#### Analysis of calcium transients

XY-T images of OGB labeled neurons were analyzed using customized Matlab (Mathworks) software. Regions of cortex were imaged at 364 × 353 pixels (42 × 41 μm) to 512 × 512 pixels (300 × 300 μm) at 0.64–1.64 s per frame. Images were realigned by maximizing the correlation between frames.

**Figure 3:**
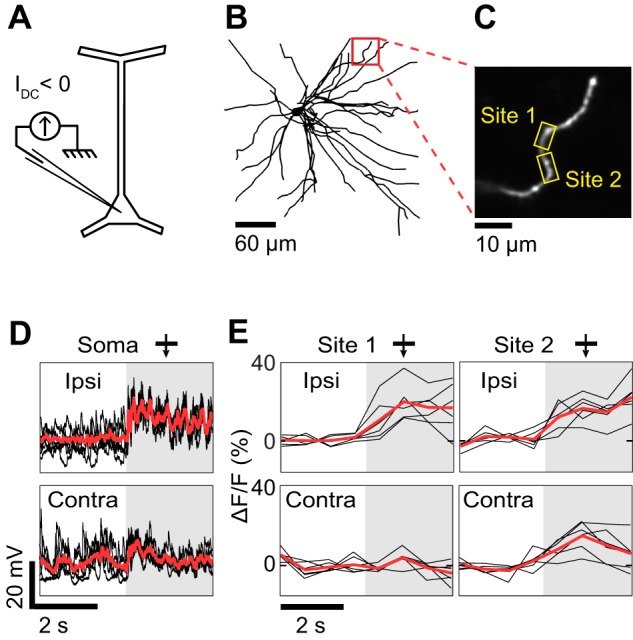
**Dendritic visual responses during complete spike suppression by current injection. (A)** Spikes were suppressed by injecting hyperpolarizing current (−0.5 nA). **(B)** Z-projection of the reconstructed somato-dendritic tree. The soma was located 310 μm below the pia. Visual responses were measured in an oblique dendrite (red box, depth: 190 μm below the pia). **(C)** Imaged dendrite. **(D)** Membrane potential responses to monocular visual stimuli presented through the ipsilateral (top trace) and the contralateral eye (bottom trace). Note the absence of spikes. **(E)** Dendritic calcium responses to stimulation of the ipsi- and contralateral eye for the two sites shown in **(C)** (yellow boxes). Shaded areas in **(D)** and **(E)** correspond to periods of visual stimulation. Responses shown in panels **(D)** and **(E)** were recorded simultaneously. Thick red trace in each panel is the average response to six repeats and black traces are individual trials. The monocular stimuli presented to the contra- and ipsilateral eyes had identical direction (270°) and spatial frequency (0.125 cycles/°).

**Figure 4:**
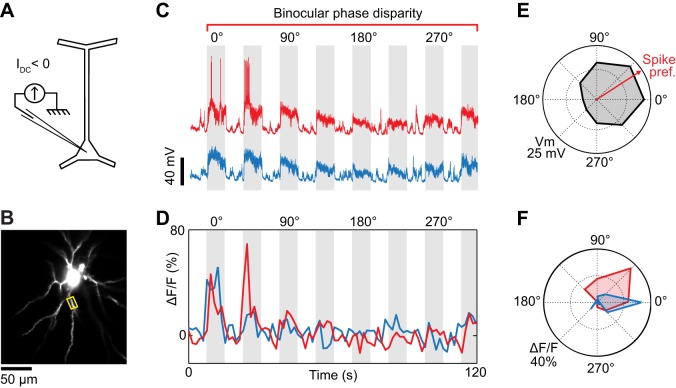
**Dendritic visual responses during partial spike suppression by current injection. (A)** Spikes were suppressed by injecting a current of −0.8 nA. **(B)** Imaging site showing the soma and basal dendrites located 345 μm below the pia. **(C)** Intracellular responses to two consecutive repeats of visual stimuli varying in binocular phase disparity. The negative current was sufficient to prevent spikes during one of the repeats (blue trace) but not the other (red trace, see leakage spikes at 0° and 45°). **(D)** Calcium responses recorded in the basal dendrite highlighted in **(B)** (yellow box). The color code (red and blue traces) corresponds to the membrane potential responses shown in **(C)**. **(E)** Polar plot of the membrane potential tuning to binocular disparity phase. Black trace: average of the two membrane potential responses shown in **(C)**; the spikes were filtered out. The red arrow indicates the preferred binocular phase disparity of the spiking response. **(F)** Polar plots of the dendritic tuning to binocular disparity phase in the absence (blue) and presence (red) of spikes.

**Figure 5:**
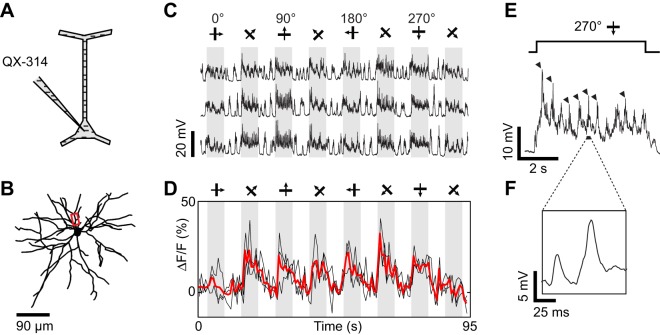
**Dendritic visual responses during pharmacological blockade of spikes in a neuron loaded with QX-314. (A)** Spikes were suppressed by QX-314 (100 mM) contained in the intra-electrode solution. **(B)** Z-projection of the reconstructed somato-dendritic tree. The soma was located 265 μm below the pia, and visual responses were imaged in a basal dendrite (red box, depth: 275 μm below the pia). **(C)** Membrane potential responses to eight stimulus directions. **(D)** Corresponding calcium responses imaged in the dendrite highlighted in **(B)**. Thick red trace: average of three repeats. **(E)** Expanded time course of the membrane potential response to the preferred stimulus direction. Each cycle of the drifting grating visual stimulus elicited QX-insensitive spikelets (arrowheads). **(F)** Expanded time course showing the shape of the QX-insensitive spikelets, which are smaller and slower than sodium spikes. No current was injected during this recording.

**Figure 6:**
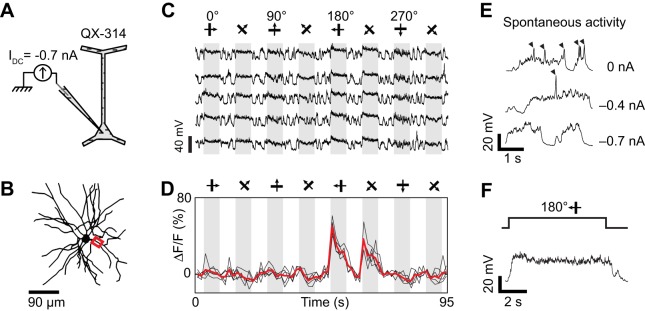
**Dendritic visual responses during pharmacological blockade of spikes in a neuron loaded with QX-314 and simultaneous injection of hyperpolarizing current. (A)** 50 mM QX-314 was included in the sharp micropipette, and −0.7 nA current was injected. **(B)** Z-projection of the reconstructed somato-dendritic tree. The soma was located 280 μm below the pia. Visual responses were imaged in an apical dendrite (red box, depth: 185 μm below the pia). **(C)** Membrane potential responses to visual stimuli moving in eight different directions (five repeats). I_DC_ = −0.7 nA. Note the absence of spikes. **(D)** Corresponding visual responses in the apical dendrite highlighted in **(B)**. Thick red trace: average of five repeats. **(E)** Spontaneous membrane potential activity for different amplitudes of constant negative current. QX-insensitive spikelets are progressively suppressed as the neuron is further hyperpolarized with −0.4 to −0.7 nA current injection. **(F)** Expanded time course of the membrane potential response at the preferred direction during negative current injection (−0.7 nA). Note the absence of QX-insensitive spikelets.

**Figure 7:**
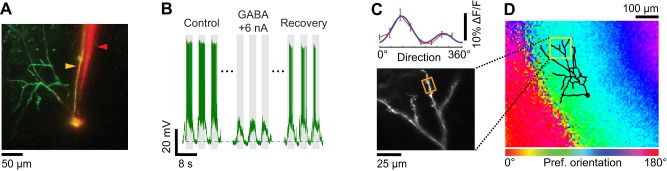
**Dendritic visual responses when spikes were prevented by GABA iontophoresis from an electrode located close to the soma. (A)** Z-projection of a layer 2/3 pyramidal neuron loaded with OGB-1. The soma was located 390 μm below the pia. The surface of the cortex was not horizontal, which in the Z-projection gave the appearance that the dendritic tree was asymmetrical and located to the left of the soma. The apical dendrites extend well beyond the 300 × 300 μm XY region shown that was available for imaging with the 40× objective. Since the cell body was located relatively deep in the cortex (390 μm) basal dendrites are not visible in the Z-projection. The sharp microelectrode (yellow arrowhead) also included Alexa 633. The iontophoresis electrode (red arrowhead) was loaded with GABA and Alexa 594. The tip of the iontophoresis electrode was located ~15 μm away from the soma. **(B)** Membrane potential responses to the preferred stimulus direction (90°) before, during, and 5 sec after GABA iontophoresis. Extracellular iontophoresis of GABA (+6 nA) combined with −0.1 nA of current delivered intracellularly was sufficient to prevent spiking. The down state Vm in the absence of visual stimulation and GABA iontophoresis was −70.5 mV (dashed line). **(C)** Direction selectivity (top panel) of an apical dendrite segment indicated by an orange box in the lower panel (depth: 110 μm below the pia; distance from cell body: 330 μm). Red trace: direction tuning curve (average of three repeats, error bars: SEM); blue trace: Gaussian fit. Spikes were suppressed by GABA iontophoresis (+10 nA, not shown). **(D)** Z-projection of the reconstructed somato-dendritic tree, superimposed on the orientation map obtained by intrinsic signal optical imaging.

**Figure 8:**
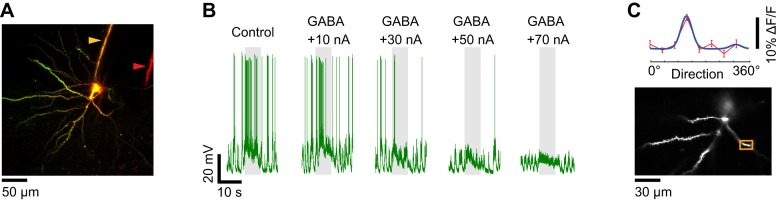
**Dendritic visual responses when spikes were prevented by GABA iontophoresis from an electrode located far from the soma. (A)** Z-projection of a layer 2/3 pyramidal neuron loaded with OGB-1. The soma was located 250 μm below the pia. The distance between the sharp electrode which included Alexa 633 (yellow arrowhead) and the iontophoresis electrode loaded with GABA and Alexa 594 (red arrowhead) is 80 μm. **(B)** Membrane potential responses of the neuron shown in **(A)** to the preferred direction (180°) before and during GABA iontophoresis at different current levels. +70 nA was necessary to prevent spiking. **(C)** Direction selectivity (top panel) of an apical dendrite segment indicated by an orange box in the lower panel (depth: 180 μm below the pia). Red trace: direction tuning curve (average of four repeats, error bars: SEM); blue trace: Gaussian fit. Spikes were suppressed by GABA iontophoresis (+70 nA, not shown).

For OGB-1 AM bulk-loading experiments, cell bodies were identified automatically through a series of morphological filters that defined the contours of cell bodies based on intensity, size and shape. Dendritic segments were initially ~2 μm in size, selected manually. Based on the similarity of response selectivity in adjacent dendritic segments, in subsequent iterations of the analysis we increased the size of each segment up to 25 μm. In a given iteration, all segments had the same size. For the purposes of comparing the three methods of spike suppression, only a small representative subset of visually responsive dendritic segments are shown in Figures 3–8.

Time courses of calcium transients were extracted by calculating mean pixel values within cell bodies or dendritic segments. The fluorescence during visual stimulation was divided by the average fluorescence during the 4–8 s baseline period prior to each stimulus to obtain the visually evoked change in fluorescence (ΔF/F). Visually responsive cell bodies or dendritic segments were defined by ANOVA across blank and *n* visual stimuli (*p* < 0.01). Cell bodies or dendritic segments selective for particular stimuli were defined by ANOVA across *n* stimulus periods (*p* < 0.01).

#### Spatial extent of suppression during GABA iontophoresis

**Figure 9:**
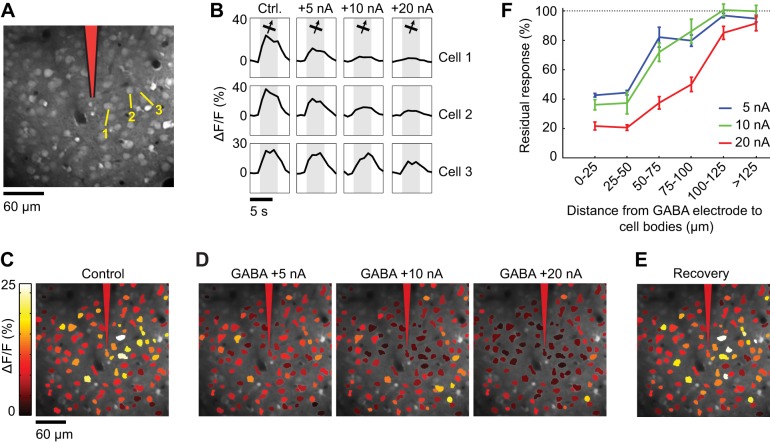
**Spatial extent of suppression during GABA iontophoresis. (A)** Calcium indicator loading in cells 230 μm below the pia. After bulk loading of OGB-1 AM, an iontophoresis electrode was inserted at the position indicated in red. **(B)** Calcium responses to the preferred direction of motion in three neuronal cell bodies (labeled in **A**) in the absence of GABA (Ctrl.) and during GABA iontophoresis. Each trace is the average response to 10 repeats. More current is required to suppress the responses as the distance between the electrode and the neuron increases. **(C)** Cell map showing the average response to the preferred direction (65°) before GABA iontophoresis. **(D)** Average response to the preferred direction during GABA iontophoresis at different current levels. **(E)** Recovery of somatic responses 20 min after cessation of GABA iontophoresis. **(F)** Residual response (see “Materials and Methods”) at different current amplitudes, as a function of the distance between the GABA iontophoresis electrode and neuronal cell bodies (mean ± SEM).

**Figure 10:**
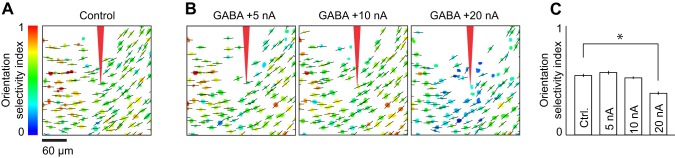
**Cortical orientation maps during application of different levels of GABA iontophoretic current. (A)** Preferred orientation (indicated by black bars) and orientation selectivity index (OSI, indicated by color scale) of each neuron in an imaging site located 260 μm below the pia. **(B)** Preferred orientation and OSI during iontophoresis of GABA at +5, +10, and +20 nA. **(C)** Mean OSI across neurons before (Ctrl.) and during iontophoresis of GABA at +5, +10, and +20 nA. Error bars: SEM, ^*^p < 0.05. In each of the four conditions shown, the superimposed cell-based maps for OSI and preferred orientation were derived from responses to the presentation of stimuli at eight directions of motion (45° step size). Each map is the average of four repeats.

An iontophoresis electrode was positioned in the center of a region of layer 2/3 cortex that was bulk-loaded with OGB-1 AM. In the first control, somatic calcium indicator responses to gratings drifting in one direction were measured before, during and after GABA iontophoresis at varying current intensities (Figure 9). The residual response in each cell was quantified as (R_GABA_/R_control_) × 100, where R_control_ and R_GABA_ are the responses before and during GABA iontophoresis, respectively. This ratio is sensitive to noise in very weakly responsive cells. Thus, when plotting the residual response as a function of distance from the GABA electrode (Figure 9F), visually responsive cell bodies that showed no significant difference in the presence and absence of GABA (*t*-test, *p* < 0.05) were scored 100 (no effect). In a second control, eight directions of drifting gratings were presented to assess the orientation selectivity index (OSI) of each cell in the presence and absence of GABA (see Figure 10):

$$\text{OSI}=\;\frac{\sqrt{{\left(\sum R\left(\theta \right)\sin\left(2\theta \right)\right)}^{2}+{\left(\sum R\left(\theta \right)\cos\left(2\theta \right)\right)}^{2}}}{\sum R\left(\theta \right)}$$

where *R*(θ) is the response at stimulus orientation θ.

### Intrinsic signal optical imaging of orientation maps

Intrinsic signals were recorded as described elsewhere (O'Herron et al., 2012). Briefly, the cortical surface was illuminated with red light (630 nm) while the animal viewed stimuli of varying orientations (5 s duration interspersed with 14 s long blanks, 8 directions of motion, 4–8 repeats). The reflected light was captured using an air objective (Olympus UPLFLN 4×, 0.13 NA, 17 mm WD) focused 600 μm below the surface and recorded by a CCD camera (Adimec-1000) mounted on the two-photon microscope. False color orientation maps were generated in Matlab by vector averaging the responses at each pixel in the region of interest.

## Results

Sharp electrodes were used to record the membrane potential and infuse OGB-1 salt in 31 pyramidal neurons in layer 2/3 of the cat visual cortex. In 16 of these neurons, recording durations were long enough to measure the membrane potential while imaging visually evoked responses on dendrites (median recording duration: 80 min, range: 25–200 min). In a given neuron, visually evoked spikes were suppressed by one of the following strategies: (1) hyperpolarization by injection of a constant negative current (*n* = 5 neurons); (2) blockade of voltage-sensitive sodium channels by including QX-314 in the electrode solution (*n* = 7 neurons); or (3) GABA iontophoresis near the soma of the intracellularly recorded neuron from an iontophoresis electrode glued to the sharp electrode (*n* = 4 neurons).

### Spike suppression by negative current injection

The effectiveness of negative current injection in preventing spikes varied across neurons and depended also on the type of visual stimulus presented. The neuron shown in Figure 3 was recorded upon the presentation of monocular visual stimuli. Spikes were prevented by reducing the contrast of the gratings to 25%, and by injecting −0.5 nA of current intracellularly. At the same time, we imaged OGB-1 fluorescence in an oblique dendrite. We found that ocular dominance varied along the length of the dendrite. One segment (Site 1) responded only to stimulation through the ipsilateral eye, whereas an adjacent segment (Site 2) responded both to stimuli presented to the ipsi- or the contralateral eye (Figure 3E). Since spikes were prevented by negative current injection (see intracellular membrane potential traces in Figure 3D), the differences in ocular dominance between the two imaged sites are likely of synaptic origin. However in 2 of 5 neurons, negative current injection was insufficient to prevent spiking in response to optimal stimuli. For the neuron shown in Figure 4, visually-evoked responses were recorded by presenting optimal gratings varying in binocular phase disparity (contrast 50%). At the preferred disparity phase, spiking was prevented during some repeats but not during others (compare membrane potential traces in blue and red, Figure 4C). As a result, the disparity phase preference of the imaged basal dendrite shifted from 0° in the absence of spikes, toward 45°, the preferred phase of the spike responses (blue vs. red in Figures 4D,F). Moreover, all eight dendritic segments examined had the same preferred phase disparity when spikes leaked but a wider range of preferred disparities when no spikes occurred (data not shown). Thus, as seen in the mouse neocortex (Jia et al., 2010), spikes back-propagating from the soma can invade the basal dendrites of cat layer 2/3 pyramidal neurons and mask the functional properties of the local dendritic inputs. We therefore investigated pharmacological strategies to prevent spiking.

### Spike suppression by intracellular QX-314

QX-314 is a membrane-impermeant lidocaine derivative which blocks voltage-gated sodium channels when applied intracellularly (Connors and Prince, 1982). In the cat visual cortex, we found that 100 mM QX-314 blocked sodium spikes in the recorded neurons (0 of 2, 1 of 2, and 3 of 3 neurons suppressed with 20, 50, and 100 mM QX-314, respectively). In these neurons where sodium spikes were blocked, stimuli moving in different directions still evoked large membrane depolarizations (Figure 5). While recording the membrane potential of the neuron, we also measured changes in calcium indicator fluorescence in basal dendrites, which showed responses to a broad range of orientations and directions (Figure 5D). However visual stimuli also evoked QX-insensitive spikelets with amplitude (10–20 mV) and duration (10–20 ms) similar to dendritic calcium spikes previously observed in layer 2/3 pyramidal neurons (Figures 5E,F, compare with Hirsch et al., 1995; Svoboda et al., 1997, 1999; Larkum et al., 2007). Thus, it is unclear whether the dendritic responses originated entirely from local synaptic inputs, or whether they were influenced by dendritic spikes initiated elsewhere in the somato-dendritic tree.

Dendritic spikes (QX-insensitive spikelets) would blur the functional differences between synaptic inputs to distinct dendrites and could make a dendrite appear more broadly tuned than the local synaptic drive. QX-insensitive spikelets were a common feature in neurons when using 50–100 mM QX-314 (*n* = 5 of 7 neurons tested, also see Hirsch et al., 1995; Svoboda et al., 1997). QX-314 may facilitate the generation and propagation of calcium spikes by increasing the membrane resistance at resting potentials (Stafstrom et al., 1982, 1985; Svoboda et al., 1997; Waters and Helmchen, 2006; Humeau and Lüthi, 2007). We could sometimes suppress these QX-insensitive spikelets by delivering additional negative current (also see Hirsch et al., 1995) and concomitantly obtain tuned calcium responses in dendritic segments (Figure 6). First, we found that intracellular injection of −0.7 nA current suppressed spontaneous QX-insensitive spikelets (Figure 6E). Second, when this current was applied during visual stimulation, we observed depolarizing plateau responses free of both sodium spikes and QX-insensitive spikelets (Figures 6C,F). Therefore the imaged dendritic responses were likely of synaptic origin, and informative of the orientation and direction tuning of neurons presynaptic to the recorded cell (Figure 6D). However in two other neurons, even large negative currents were insufficient to completely suppress QX-insensitive spikelets (not shown). We conclude that the potential effect of QX-314 on dendritic calcium electrogenesis may be difficult to control in some layer 2/3 neurons of the cat visual cortex.

### Spike suppression by GABA iontophoresis

Pharmacological suppression of spiking can also be attained by iontophoresis of GABA in the vicinity of the intracellularly recorded neuron. To this end we assembled double-barrel electrodes consisting of one sharp intracellular electrode protruding slightly in front of an iontophoresis electrode (Figure 2D). After the sharp electrode impaled a neuron, we measured input resistance and recorded visually evoked spiking responses (see “Materials and Methods”). Thereafter, GABA was ejected from the iontophoresis electrode. When the tips of the two electrodes were less than 20 μm apart (Figure 7A), low iontophoretic currents (+5 to +10 nA) were sufficient to rapidly, completely, and reversibly quench spiking responses to optimal visual stimuli (Figure 7B). The amount of iontophoretic current necessary to prevent spiking was further minimized by the additional delivery of a small negative current through the sharp electrode. As a result, we were able to measure the orientation tuning of inputs on dendrites (Figure 7C). The preferred orientation matched that of the local cortical region, which was measured by intrinsic signal optical imaging (Figure 7D). The very small positive extracellular current (≤10 nA) we used for ejecting GABA did not in itself produce a depolarization since the membrane potential down state was unaffected (see horizontal dotted line in Figure 7B).

When the multi-barrel electrodes were fabricated with large inter-tip distances between the sharp intracellular and GABA iontophoretic electrodes, much more iontophoretic current (+70 nA) was necessary to suppress the responses to optimal stimuli (Figure 8). The larger quantity of GABA released could affect the functional dendritic inputs to the imaged neuron (Figure 8C) by inhibiting other neurons located in the vicinity of the tip of the iontophoresis electrode. Therefore it was essential to determine the relationship between the spatial extent of GABAergic inhibition and the intensity of the iontophoretic current.

In two separate control experiments, we labeled populations of layer 2/3 neurons with OGB-1 AM (*n* = 2 cats). First, we presented a stimulus whose orientation was near optimal for all the imaged neurons and measured the somatic calcium response while GABA was delivered from an iontophoresis electrode identical to those used for making double-barrel electrodes (Figure 9). As expected, GABA iontophoresis suppressed responses in cell bodies that were located close to the electrode, and this suppression increased in strength and spatial extent with the amplitude of the iontophoretic current. At low current intensities (+5 to +10 nA), suppression was restricted to a radius of ~50 μm around the electrode tip; neurons located more than 100 μm away were generally not suppressed (Figure 9). Second, we found that +5 to +10 nA GABA iontophoretic currents had little effect on the structure of the cortical orientation map (Figure 10). The preferred orientation across the imaged neurons was not significantly different before and during GABA iontophoresis (Kruskal–Wallis test, *p* = 0.13, *n* = 110 selective neurons). Orientation selectivity across the population was significantly decreased by GABA iontophoresis at +20 nA, but not at +5 nA and +10 nA (Figure 10C, Kruskal–Wallis and Tukey tests, *p* < 0.05, *n* = 130 responsive neurons).

## Discussion

We have shown that the location and selectivity of sensory-evoked synaptic inputs can be measured by changes in calcium indicator fluorescence on dendrites in the cat visual cortex. Subcellular mapping of functional inputs was possible only when action potentials in the recorded neuron were prevented, which we verified by intracellular sharp electrode recording. We compared different strategies to prevent spiking, and found that GABA ejection from a perisomatic iontophoresis electrode provided the best control. To our knowledge this is the first time that functional inputs have been mapped on the dendritic tree of cortical neurons in a non-rodent mammal.

The development of dendritic imaging using fluorescent calcium indicator proteins and synthetic dyes *in vivo* holds great promise for the study of cortical functional connectivity (see Grienberger and Konnerth, 2012 for a review). Single-cell loading of synthetic calcium indicators was first achieved with sharp intracellular electrodes (*in vitro*: Jaffe et al., 1992, *in vivo*: Svoboda et al., 1997). However the first successful functional imaging studies of dendritic synaptic inputs *in vivo* relied on whole-cell recording through patch electrodes (Jia et al., 2010; Chen et al., 2011; Varga et al., 2011). One advantage of the whole-cell patch electrode is that it offers good electrical access to the cell's interior, resulting in efficient control of neuronal activity. Moreover, the position of the electrode in the cortex can be adjusted to label a targeted neuron (Margrie et al., 2003; Nevian and Helmchen, 2007; Kitamura et al., 2008).

Compared to patch electrodes, sharp electrodes are much thinner, and therefore much more flexible. This flexibility provided good intracellular recording stability in the cat, after we dampened brain pulsations by covering the craniotomy with agarose and a coverglass. The time required for labeling neurons was comparable to the loading times reported previously in sharp and patch intracellular recordings (Svoboda et al., 1997, 1999; Helmchen et al., 1999; Jia et al., 2011). Finally, since the dye does not accumulate in the extracellular space as the sharp electrode is advanced inside the neocortex, intracellular recordings can be attempted many times with the same electrode at a given cortical site. Thus, sharp recordings may provide higher rates of success for simultaneous intracellular recording and two-photon dendritic imaging in large experimental preparations.

Hyperpolarization by current injection through the sharp electrode is the most straightforward strategy to control spiking in the recorded neuron. In practice however, the maximum negative current our sharp electrodes can deliver is approximately −1 nA. This is seldom sufficient to completely prevent spikes in response to optimal binocular stimuli, which evoke a large excitatory drive in cat cortical neurons (Freeman and Ohzawa, 1992; Kara and Boyd, 2009). The current needed to block all spikes may be reduced by presenting low contrast stimuli or by mapping the receptive fields with sparse or dense noise, but the dendritic calcium signals would also be correspondingly weaker.

QX-314 is an appealing pharmacological alternative to current injection for blocking spikes (also see Takahashi et al., 2012), but it also has drawbacks. First, its action is fast and irreversible, thus precluding the comparison between functional inputs, i.e., synaptic/dendritic responses, and output, i.e., spiking responses. Second, QX-314 does not only block the voltage-gated sodium channels involved in spike generation; it also affects other sodium, potassium, and calcium conductances (Stafstrom et al., 1982, 1985; Andreasen and Hablitz, 1993; Perkins and Wong, 1995; Talbot and Sayer, 1996). Intracellular infusion of QX-314 increases the membrane resistance of neocortical pyramidal neurons (Stafstrom et al., 1982, 1985; Waters and Helmchen, 2006). This in turn may decrease the threshold for calcium spikes and facilitate their propagation across the dendritic tree (Svoboda et al., 1997; Paré and Lang, 1998; Humeau and Lüthi, 2007). Additional studies are needed to establish how QX-insensitive spikelets and their suppression by current injection depend on cell type and sub-laminar location.

Multi-barrel electrodes are typically used to study the *extracellular* responses to sensory stimuli during the iontophoretic administration of various pharmacological agents (Stone, 1985; Kara and Friedlander, 1999; Katzner et al., 2011). Double-barrel electrodes for simultaneous *intracellular* recording and local iontophoresis *in vivo* are much less common (but see Douglas and Martin, 1991; Frégnac and Shulz, 1999; Ego-Stengel et al., 2002; Ferron et al., 2009). We found that intracellular recordings *in vivo* were not more difficult to obtain with the double-barrel electrode, compared to the conventional single sharp electrode. Moreover, of the three spike suppression methods tested, local GABA iontophoresis offered the finest control such that the spiking responses to optimal binocular stimuli could be completely prevented with +5 to +10 nA of iontophoretic current. Our results and an earlier study (Ferron et al., 2009) suggest that the prevention of action potentials by local GABA micro-iontophoresis occurs primarily by shunting the membrane conductance with a minimal change in the resting membrane potential. These data are consistent with the high levels of expression of GABA_A_ receptors on the cell body and axon initial segment of cortical pyramidal neurons (Douglas et al., 2003). Moreover, unlike QX-314, suppression by GABA iontophoresis was reversible such that within a matter of minutes from switching from ejection to retention current, the visual response recovered completely. In addition, other types of regenerative potentials, e.g., dendritic spikes, were also absent during GABA iontophoresis, ensuring that the calcium responses we observed originated from local synapses.

Our double-barrel electrodes were fabricated such that the tip of the iontophoresis electrode was located close to the soma of the recorded neuron, enabling GABAergic inhibition of spikes at low current intensities. Delivering additional hyperpolarizing current intracellularly through the sharp electrode further minimized the GABA iontophoretic current that was needed to prevent all spikes. Most of the neurons suppressed by +5 to +10 nA GABA iontophoretic currents were located less than 50 μm away from the electrode tip. This corresponds to approximately 30 neurons (Beaulieu and Colonnier, 1983), of which ~10% are likely to send inputs to the soma and basal dendrites of the imaged neuron (Thomson et al., 2002). Thus, just a few of the many thousands of excitatory synaptic inputs on the imaged recorded neuron will be turned off by +5 to +10 nA GABA iontophoretic currents. Other neurons, whose dendrites cross into the region where GABA is released, may also become less responsive, but our data indicates that their tuning properties should be preserved: indeed with +5 to +10 nA GABA iontophoresis we did not observe any significant effect on the structure of the local orientation map and the OSI of the local population of neurons (Figure 10). In addition, the calcium influx in dendrites associated with synaptic activation of spines (Chen et al., 2011) may also be reduced in the vicinity of the iontophoresis electrode, but is unlikely to be vetoed because the vast majority of inhibitory synapses on dendrites are located on shafts, not spines (Douglas et al., 2003). Overall, these data and earlier studies suggest that while very local micro-iontophoretic application of GABA may affect the amplitude of the calcium influx at a small minority of the imaged synapses, their selectivity is unlikely to be affected. We do not recommend the use of GABA iontophoretic currents of +20 nA or higher because they distort local functional maps and selectivity (Figures 10B,C).

As a future development of our technique, intracellular recordings may not be required for dendritic functional imaging provided that the iontophoretic current has been calibrated for spike suppression in a particular preparation. Neurons could be sparsely labeled by electroporation or by a genetically encoded calcium indicator, e.g., GCaMP6 (Chen et al., 2012), and their spikes suppressed by GABA iontophoresis from a single electrode brought close to the labeled soma.

In conclusion, the choice of patch vs. sharp electrodes for dendritic functional imaging with synthetic dyes depends on the experimental model being used. In small animals such as rodents, patch electrodes are preferable, due to their lower impedance. In large mammals such as the cat, sharp electrodes may be more suitable, because they provide long-duration intracellular recordings and because the dye does not accumulate in the extracellular space as the pipette advances to the neuron. More generally, by providing a means to apply pharmacological agents such as neuromodulators or channel blockers *in vivo* while simultaneously imaging calcium responses in dendrites, double-barrel electrodes could advance our understanding of the role of synaptic plasticity and dendritic excitability during sensory processing.

### Conflict of interest statement

The authors declare that the research was conducted in the absence of any commercial or financial relationships that could be construed as a potential conflict of interest.
